# Genomic Profiling of Antibiotic-Resistant *Escherichia coli* Isolates from Surface Water of Agricultural Drainage in North-Western Mexico: Detection of the International High-Risk Lineages ST410 and ST617

**DOI:** 10.3390/microorganisms10030662

**Published:** 2022-03-20

**Authors:** José Antonio Magaña-Lizárraga, Bruno Gómez-Gil, José Guadalupe Rendón-Maldonado, Francisco Delgado-Vargas, Inés Fernando Vega-López, María Elena Báez-Flores

**Affiliations:** 1 Unidad de Investigaciones en Salud Pública “Dra. Kaethe Willms”, Facultad de Ciencias Químico Biológicas, Universidad Autónoma de Sinaloa, Ciudad Universitaria, Culiacan 80013, Mexico; jantonio.mlga@gmail.com (J.A.M.-L.); jgrendonm@uas.edu.mx (J.G.R.-M.); fdelgado@uas.edu.mx (F.D.-V.); 2 Centro de Investigación en Alimentación y Desarrollo, A. C. (CIAD), Unidad Mazatlán en Acuicultura y Manejo Ambiental, AP.711, Mazatlan 82112, Mexico; bruno@ciad.mx; 3 Parque de Innovación Tecnológica, Universidad Autónoma de Sinaloa, Culiacan 80013, Mexico; ifvega@uas.edu.mx

**Keywords:** multidrug-resistance, environmental *E. coli*, aquatic environment, high-risk *E. coli* clones, ETEC O6, class 1 integron, whole-genome sequencing, agricultural drainage

## Abstract

Aquatic environments are recognized as one of the main reservoirs for the emergence and dissemination of high-risk lineages of multidrug-resistant (MDR) bacteria of public health concern. However, the genomic characteristics of antibiotic-resistant *Escherichia coli* isolates from aquatic origins remain limited. Herein, we examined the antibiotic resistance and virulence genomic profiles of three *E. coli* recovered from surface water in northwest Mexico. Antimicrobial susceptibility testing, whole-genome sequencing (WGS), and in-depth in silico analysis were performed. Two *E. coli* exhibited MDR phenotypes. WGS-based typing revealed genetic diversity, and phylogenetic analysis corroborated a notable divergent relationship among the studied *E. coli*. One *E. coli* strain, harboring enterotoxigenic and extraintestinal pathogenic-associated virulence genes, was assigned to the ST4 lineage. MDR *E. coli*, belonging to the international high-risk clones ST410 and ST617, carried genes and mutations conferring resistance to aminoglycosides, β-lactams, quinolones, sulfonamides, tetracyclines, and trimethoprim. This study describes, for the first time, the detection and genomic profiling of high-risk lineages of *E. coli* ST410 and ST617 from surface water in Mexico. Additionally, our results underscore the role of surface water as a reservoir for critical pathogenic and MDR *E. coli* clones and the need for the surveillance and monitoring of aquatic environments via WGS from the One Health perspective.

## 1. Introduction

Antimicrobial resistance (AMR) has been a primary global public health issue in recent decades. AMR hinders clinical effectiveness and limits the therapeutic options for infectious diseases, leading to increased healthcare costs, treatment failure, morbidity, and mortality rates. At present, the estimated AMR-related death toll accounts for 700,000 annually, and it is projected to increase to 10 million deaths per year by 2050 unless action is taken [1]. AMR phenomenon represents a complex and multifaceted threat of growing concern to the human, animal, and environmental health due to the emergence, persistence, and spread of antibiotic resistance genes (ARGs) and their acquisition by clinically relevant bacteria in each interconnected sector [2].

The importance of the environment as a reservoir of antibiotic resistance and its role in the spread of potential ARGs has been widely recognized. Antibiotic resistance can arise either by mutational mechanisms or by resistance-conferring gene acquisition via horizontal gene transfer (HGT) events, that is, conjugation, transformation, or transduction. HGT is considered the most important factor in the recruitment and transmissibility of ARGs from the environmental gene pool to pathogenic bacteria [3].

Among all environmental compartments, the aquatic ecosystems are constantly subjected to anthropogenic contamination with antibiotics through the direct or indirect discharges of agricultural, aquaculture, domestic, hospital, and industrial effluents. The selective pressure exerted by antibiotic contamination impacts the resident bacterial communities towards the selection of antibiotic-resistant bacteria (ARB) and enrichment of drug-resistant genetic determinants [4]. Furthermore, aquatic environments have dynamic and distinct microbial composition patterns influenced by temporal and spatial disparities in physiochemical and biotic factors, including environmental stresses and nutrient composition [5]. Consequently, the aquatic milieus constitute an ideal reservoir for microbial genetic exchange, evolution, and dissemination of ARGs and ARB.

Among the ARB harboring transmissible resistance traits of greatest clinical relevance are extended-spectrum β-lactamase (ESBL)-producing *Enterobacteriaceae*, which have become a challenge for infection control because of their worldwide endemicity and multidrug-resistant (MDR) phenotypes [6,7]. *Escherichia coli* (*E. coli*) is a highly versatile member of the *Enterobacteriaceae* family that constitutes an important component of the commensal gastrointestinal microbiota of warm-blooded animals, including humans. However, its genome plasticity and HGT mechanisms have led to the evolution of this microorganism into highly adapted antibiotic-resistant and pathogenic clones [8].

Depending on the site of infection, pathogenic *E. coli* strains are classified as intestinal pathogenic *E. coli* (InPEC) or extraintestinal pathogenic *E. coli* (ExPEC) and further sub-categorized into distinct pathotypes based on clinical disease manifestations, virulence factors (VFs), and genetic background [9,10]. The long-term survival of *E. coli* has been described in secondary habitats, including food products, sediments, soil, and water [11,12]. Thus, environmental *E. coli* strains could be a source of opportunistic and MDR-related diseases of public health significance in animals and humans; therefore, such strains must be monitored and tracked.

Several studies have identified antibiotic-resistant and pathogenic *E. coli* strains in environmental water samples from Mexico [13,14,15,16,17]. Previous studies in Sinaloa, Mexico, have reported *E. coli* as a major biological pollutant of diverse aquatic resources, including agricultural drainage, drinking, and irrigation water [18,19,20]. Canizalez-Roman et al. [21] demonstrated multidrug-resistant InPEC strains in surface water samples from irrigation channels and river water across Sinaloa State, where they exhibited resistance to β-lactams, tetracycline, and trimethoprim/sulfamethoxazole.

To the best of our knowledge, genomic data describing the mechanisms underlying antibiotic resistance and virulence properties of *E. coli* from environmental water samples in Mexico are extremely limited. Therefore, this study aimed to examine, from a genomic perspective, the antibiotic resistance patterns and virulence determinants of *E. coli* isolates recovered from surface water of an agricultural drainage ditch that constantly receives irrigation water and domestic wastewater. In this study, we describe the genomic characteristics and determine the genetic diversity regarding the virulence-associated and antimicrobial resistance genes, plasmids, and prophage content of three antibiotic-resistant *E. coli* strains from the aquatic environment, two of which are international high-risk clones.

## 2. Materials and Methods

### 2.1. Bacterial Isolation and Identification

*E. coli* isolates ADD147, ADD167, and ADD183 were recovered from surface water samples in a longitudinal water quality assessment of a section (3.6 km) of the agricultural drainage ditch known as “La Michoacana” (30 km in total length) in the Sinaloa Valley (Navolato Municipality, Sinaloa, Mexico) during 2013. Overall, the microbiological and physicochemical water characteristics prevailing during sampling were in the following ranges for total coliforms (TC) (2.86 × 10^4^−3.23 × 10^7^ CFU/mL), fecal coliforms (FC) (5.45 × 10^3^–1.77 × 10^7^ CFU/mL), pH (7.8–8.01), temperature (21.51–24.76 °C), dissolved oxygen (DO) (8.65–21.24%), and salinity (0.54–0.64) [20]. The ditch has a semicircular profile (4 m wide × 2 m deep) on a plane landscape surrounded by crop fields and receives untreated agricultural and domestic wastewater discharge [22]. Bacterial isolation and species identification were conducted as previously described by Ahumada-Santos et al. [20]. All collected *E. coli* isolates were stored in brain heart infusion (BHI) broth containing 8% (*v*/*v*) DMSO at −80 °C. *E. coli* ADD147, ADD167, and ADD183 were selected based on preliminary phylogroups typing, antimicrobial resistance, and virulence profiles observed in testing for further study.

### 2.2. Antimicrobial Susceptibility Testing (AST)

The phenotypic antimicrobial resistance profile of *E. coli* isolates was determined using the disk diffusion method following the Clinical and Laboratory Standards Institute (CLSI) guidelines [23]. Twelve commercial antibiotic disks obtained from Diagnostic Research (ID), Mexico (PT-35 reference) spanning several antimicrobial classes were tested: aminoglycosides (amikacin (AK; 30 µg); gentamicin (GE; 10 µg); netilmicin (NET; 30 µg)), β-lactams (ampicillin (AM; 30 µg), carbenicillin (CB; 100 µg), cephalothin (CF; 30 µg), cefotaxime (CFX; 30 µg)), fluoroquinolones (ciprofloxacin (CPF; 5 µg), norfloxacin (NOF; 10 µg)), nitrofurans (nitrofurantoin (NF; 300 µg)), phenicols (chloramphenicol (CL; 30 µg)), and folate pathway antagonists (trimethoprim/sulfamethoxazole (SXT; 1.25/23.75 µg)). *E. coli* ATCC 25922 was used as a quality control strain. The inhibition zones were measured and interpreted according to the CLSI breakpoint criteria (M100 ED31:2021) [24]. The categorical interpretation of intermediate susceptibility was described as not susceptible. The isolates were classified as MDR strains if they exhibited non-susceptibility to at least one antimicrobial agent in three or more antimicrobial classes [25].

### 2.3. DNA Extraction, Whole-Genome Sequencing (WGS), and Read Preprocessing

For DNA extraction, each *E. coli* isolate was grown in Luria-Bertani (LB) broth at 37 ± 2 °C for 24 h. Genomic DNA (gDNA) was extracted using the ZymoBIOMICs DNA Miniprep Kit (Zymo Research Corp., Irvine, CA, USA) according to the manufacturer’s protocol. The purity, concentration, and integrity of gDNA were assessed by spectrophotometry (Nanodrop Lite, Thermo Fisher Scientific, Waltham, MA, USA) and electrophoresis on a 1.0% agarose gel. Paired-end DNA libraries were prepared using the Nextera XT Library Preparation Kit (Illumina, San Diego, CA, USA) following the manufacturer’s instructions, and sequenced on the Illumina MiniSeq platform with a 2 × 150 bp approach. Sequence data quality was evaluated using the FastQC software v.0.11.9 [26]. Raw sequencing reads were quality trimmed and filtered using Cutadapt v2.4 [27] to remove adapters, low-quality bases (<Q30), and short reads (<50 bp). The processed reads were submitted to the KmerFinder v3.2 [28] web tool from the Center for Genomic Epidemiology (CGE) (http://www.genomicepidemiology.org/, accessed on 20 November 2021) for species confirmation.

The raw genome data were submitted to the Sequence Read Archive (SRA) database of the National Center for Biotechnology Information (NCBI) under BioProject PRJNA715781 (https://www.ncbi.nlm.nih.gov/bioproject/PRJNA715781, accessed on 19 March 2021) and are accessible through individual SRA accession numbers: SRX10394959 (ADD147), SRX10394960 (ADD167), and SRX10394961 (ADD183).

### 2.4. Genome Assembly and Annotation

Following quality control and trimming, high-quality reads were assembled into contigs using SPAdes v3.15.1 [29] employing the “-- careful” option and different k-mer lengths (21, 33, 55, 77, 99, 107, and 117) and evaluated with QUAST v5.0.2 [30]. Due to the presence of small contigs after de novo assembly, a reference-guided scaffolding was performed using as a reference the best matching output from the KmerFinder tool. Therefore, the MeDuSa software [31] and the genome sequence of *E. coli* YD786 (RefSeq accession: GCF_001442495.1), WCHEC005784 (RefSeq accession: GCF_003051985.2), and 2014EL-1346-6 (RefSeq accession: GCF_002741195.1) were used for genome scaffolding of *E. coli* ADD147, ADD167, and ADD183, respectively. Scaffolds with lengths of less than 300 bp were excluded. The annotation was performed using Prokka v1.14 [32].

### 2.5. Serotyping, Phylogrouping, Multilocus Sequence Typing (MLST), Virulence, and Antimicrobial Resistance-Associated Genes

To perform the in silico molecular characterization, the genomes of *E. coli* sequenced here were analyzed using web tools provided by the CGE under default setting parameters (accessed on 21 November 2021). The O and H serotypes were defined using SerotypeFinder v2.0 [33]. The sequence type (ST) was determined following the seven-locus scheme proposed by Achtman [34] using MLST v2.0 (*Escherichia coli* #1 option) [35]. Virulence-associated genes (VAGs) and chromosomal point mutations along with acquired antimicrobial resistance determinants were identified and annotated with VirulenceFinder v2.0 [36] and ResFinder v4.1 [37], respectively. In addition, *E. coli* phylogroup classification based on the Clermont et al. scheme [38] was assessed using the ClermonTyping web tool [39] (http://clermontyping.iame-research.center/index.php, accessed on 21 November 2021).

### 2.6. Plasmid Replicons and Prophages Regions

Plasmid replicon identification was performed using PlasmidFinder v2.1 from the CGE using a minimum 90% nucleotide identity and 60% coverage threshold [40]. Prophage sequences in E. coli genomes were determined with Phage Search Tool Enhanced Release (PHASTER) (https://phaster.ca/, accessed on 21 November 2021). Prophage regions are predicted to be intact, questionable, and incomplete if the prophage sequence scores are ≥90, 70–90, and ≤70, respectively [41]. Only prophages identified as intact were used in the current analysis.

### 2.7. Phylogenetic Analysis

To place our three sequenced *E. coli* isolates into a global context, a collection of isolates with the same ST (i.e., ST4, ST410, and ST617) were selected from EnteroBase using the Achtman seven gene MLST scheme as a query (https://enterobase.warwick.ac.uk/species/index/ecoli, accessed on 2 March 2022) [42]. Phylogenetic analysis was performed using GrapeTree to construct a minimum spanning tree based on the core-genome MLST (cgMLST) V1 + Hierarchical Clustering (HierCC) V1 scheme from EnteroBase through the rapid neighbor-joining (RapidNJ) algorithm [43].

Because ST410, ST617, and ST4 phylogenetic trees comprised an excess of 200 isolates, refined subtrees were generated from clades containing *E. coli* ADD147, ADD167, and ADD183. Branch lengths were used to calculate the cgMLST allelic differences between closely related isolates. Metadata of *E. coli* isolates used for ST410, ST617, and ST4 subtree reconstruction are described in Supplementary Tables S1–S3, respectively.

## 3. Results

### 3.1. Genome Sequencing

*E. coli* ADD147, ADD167, and ADD183 were recovered in 2013 from the surface water of an agricultural drainage ditch and subjected to WGS. *E. coli* genomes were assembled de novo, yielding a coverage depth of 54.6× and exhibiting a genome length of 4.75 Mbp, 540 contigs, and 50.81% of GC content on average (Table 1). Because fragmented assemblies were obtained, the complete genome sequence of *E. coli* YD786 (GCF_001442495.1), WCHEC005784 (GCF_003051985.2), and 2014EL-1346-6 (GCF_002741195.1) were used as a reference to generate scaffolds. Hence, *E. coli* ADD147, ADD167, and ADD183 genome assemblies resulted in 47, 22, and 6 scaffolds, respectively (Table 1).

### 3.2. Phylogroups, Serotype, and Sequence Types

Based on the WGS data, in silico characterization was performed to gain insights into the genetic diversity of these *E. coli* isolates. In summary, two phylogenetic groups were identified. *E. coli* ADD167 and ADD183 were assigned to phylogroup A, whereas ADD147 was allocated to phylogroup C. The O:H typing predicted three dissimilar serotypes among the sequenced *E. coli* genomes, of which *E. coli* ADD147 was O-nontypable (ONT). Similarly, three distinct STs were detected, assigning *E. coli* ADD147, ADD167, and ADD183 to ST410, ST617, and ST4, respectively. Based on MLST data, both ST4 and ST617 were grouped within clonal complex 10 (CC10) (Table 1).

### 3.3. Virulence-Associated Genes (VAGs)

Whole-genome sequences were surveyed using the VirulenceFinder database (https://cge.cbs.dtu.dk/services/VirulenceFinder/, accessed on 21 November 2021) to identify the virulence potential of *E. coli*. Therefore, 14 different VAGs were detected, and the highest frequencies were for *terC* (tellurium ion resistance protein), followed by *astA* (EAST-1 heat-stable toxin), *capU* (hexosyltransferase homolog), *gad* (glutamate decarboxylase), and *iss* genes (increased serum survival protein). Each *E. coli* strain displayed a different combination of virulence genes (Table 2).

Analysis of the distribution of VAGs indicated that strains under CC10 (ADD167 and ADD183) partially shared a virulence profile. However, *E. coli* ADD183 also displayed genes involved in capsular polysaccharide biosynthesis (*kpsE*, *kpsM_K15*), increased serum survival (*traT*), toxin production (*ltcA*, heat-labile enterotoxin A subunit), and protease production (*eatA*), namely, the serine protease autotransporter of *Enterobacteriaceae* (SPATE) EatA. A key feature of the enterotoxigenic *E. coli* (ETEC) pathotype, a member of the InPEC group, is the ability to express heat-labile (LT) and/or heat-stable (ST) enterotoxins [8]. Although further exploration is required, *E. coli* ADD183 was assigned to the ETEC pathotype according to the virulence profile predicted in this study.

### 3.4. Resistance Profile and Antimicrobial Resistance Genes (ARGs)

The phenotypic antimicrobial susceptibility was determined by disk diffusion according to CLSI standards. In summary, the phenotypic resistance profiles of *E. coli* ADD147 and ADD167 were comparable and cataloged both as MDR bacteria owing to their resistance to antibiotics from the β-lactam, fluoroquinolone, and folate pathway antagonists antimicrobial classes (Table 2) [25]. Both MDR *E. coli* strains showed resistance to ampicillin, carbenicillin, ciprofloxacin, norfloxacin, and trimethoprim/sulfamethoxazole. However, *E. coli* ADD147 further exhibited resistance to chloramphenicol, gentamicin, and netilmicin, whereas *E. coli* ADD167 was resistant to first- and third-generation cephalosporins (i.e., cephalothin and cefotaxime), suggesting a presumptive ESBL phenotype. In addition, *E. coli* ADD167 was not susceptible to gentamicin and nitrofurantoin. In contrast, ADD183 was sensitive to all antibiotics except ciprofloxacin, to which it remained not susceptible (Table 2).

Genome-based prediction of antimicrobial resistance determinants confirmed the previously observed phenotypic resistance profiles. We identified 21 different acquired genetic elements associated with resistance to β-lactams, aminoglycosides, lincosamides, phenicols, tetracyclines, and trimethoprim/sulfamethoxazole (Table 2). The multidrug transporter gene *mdf(A)* was found in all studied genomes. Two β-lactamase-encoding genes were identified: TEM-1-type narrow-spectrum β-lactamase (*bla_TEM-1B_*) and CTX-M-type ESBL (*bla_CTX-M-15_*). In total, eight allelic variants encoding aminoglycoside-modifying enzymes were detected (*aadA1*, *aadA2*, *aadA5*, *aac(3)-IIa*, *aac(3)-IId*, *aph(3′)-Ia*, *aph(3″)-Ib*, *aph(6)-Id*). Sulfonamide (*sul1*, *sul2*, and *sul3*) and trimethoprim (*dfrA12* and *dfrA17*) resistance genes were present in two *E. coli* strains. Notably, the lincomycin resistance gene *lnu(F)* and the genes responsible for phenicol resistance (i.e., *floR* and *cmlA1*) were further observed in *E. coli* ADD147. In addition, two *E. coli* strains carried the genes *tet(A)* and *tet(B)* involved in tetracycline resistance. Nonetheless, neither lincosamide nor tetracycline antimicrobial classes were tested phenotypically. No carbapenem- and colistin-resistance-associated determinants were detected in the *E. coli* under study.

Further sequence analysis indicated that both MDR *E. coli* ADD147 and ADD167 possess a multidrug resistance region, which each houses a class 1 integron with ARGs associated. In strain ADD147, we identified a *sul3*-associated integron with a genetic structure *dfrA12*–*orfF*–*aadA2*–*cmlA1*–*aadA1*–*qacH*–*tnp440*–*sul3*, conferring resistance to trimethoprim, aminoglycoside, chloramphenicol, and sulfonamide, respectively. The chloramphenicol/florfenicol resistance gene *floR* was located upstream of the identified *sul3*-associated class 1 integron. In contrast, the class 1 integron found in *E. coli* ADD167 was flanked by regions co-harboring the *ISEcp1-bla_CTX-M-15_-orf477*Δ transposition unit and *aac(3)-IIa* genes (upstream), which confer resistance to β-lactams, including broad-spectrum cephalosporins and monobactams, and aminoglycosides, respectively; and the *sul2*–*aph(3″)-Ib*–*aph(6)*-*Id* gene array (downstream), involved in sulfonamide and aminoglycoside resistance. The genetic structure identified in this class 1 integron was the *dfrA17*–*aadA5*–*qacE*Δ*1*–*sul1* gene cassette.

With regard to (fluoro)quinolone resistance, no plasmid-mediated quinolone resistance (PMQR) genes, such as *qnr*, *qepA,* or *oqxAB*, were observed. However, nucleotide sequence analysis of the quinolone resistance-determining region (QRDR) revealed point mutations leading to non-synonymous substitutions in the DNA gyrase and topoisomerase IV targets. In particular, double amino acid exchange was found in GyrA (S83L, D87N), whereas the S80I and S458A substitutions were recognized in ParC and ParE, respectively (Table 2). Additional chromosomal point mutations that confer antibiotic resistance have not been identified.

### 3.5. Detection of Plasmid Replicons and Prophages

WGS-based analysis using PlasmidFinder identified seven different replicons from both large and small plasmid groups, specifically, those relative to incompatibility F and X groups, and Col-like plasmids, respectively (Table 2). *E. coli* ADD147 concomitantly hosted two IncF-type replicons, IncFIB (AP001918) and IncFIC (FII), and the IncX-type (IncX1) and Col(pHAD28) plasmid groups, whereas *E. coli* ADD167 only harbored Col-like plasmids. *E. coli* ADD183 carried IncFII (pCoo) and ColRNAI plasmid replicons. We could not associate VAGs or ARGs with putative plasmids of *E. coli* ADD147 and ADD167. The putative IncFII plasmid (~125.3 kbp) from *E. coli* ADD183 contained several virulence-associated genes, including *astA*, *capU*, *eatA*, *ltcA*, and *traT*.

PHASTER analysis of the *E. coli* genomes identified 33 phage-related sequences. However, only seven prophage regions were predicted to be intact and considered for further analysis (Table 3); these showed homology to six different phages. Their length varied from 11 to 53 kb, of which the most frequent was the *Enterobacteria* phage P88.

Predicted prophage regions were manually inspected to identify putative active phage signatures, that is, attachment site (*att*) sequences (*attL* and *attR*) and genes encoding structural proteins, DNA packaging and regulation, insertion into the host, and lysis. Prophages similar to VT2phi_272, pro483, and SH2026Stx1 lacked attachment sites, suggesting defective phages. Conversely, the prophages BP-4795, P88, and P4 possessed *att* sequences; however, the P4-like prophage cargo only contained integrase and a few structural-related proteins, lacking recombinase, terminase, transposase, and lysis module. Except for the *iss* gene encoded in the BP-4795 prophage, none of the predicted prophages carried further genetic elements implicated in virulence or antimicrobial resistance properties.

### 3.6. Phylogenetic Analysis

The EnteroBase search resulted in a total of 1522, 508, and 209 isolates available for phylogenetic analysis of *E. coli* ST410, ST617, and ST4 lineages, respectively, including ADD147, ADD167, and ADD183. All of them are derived from diverse isolation sources.

**Figure 1:**
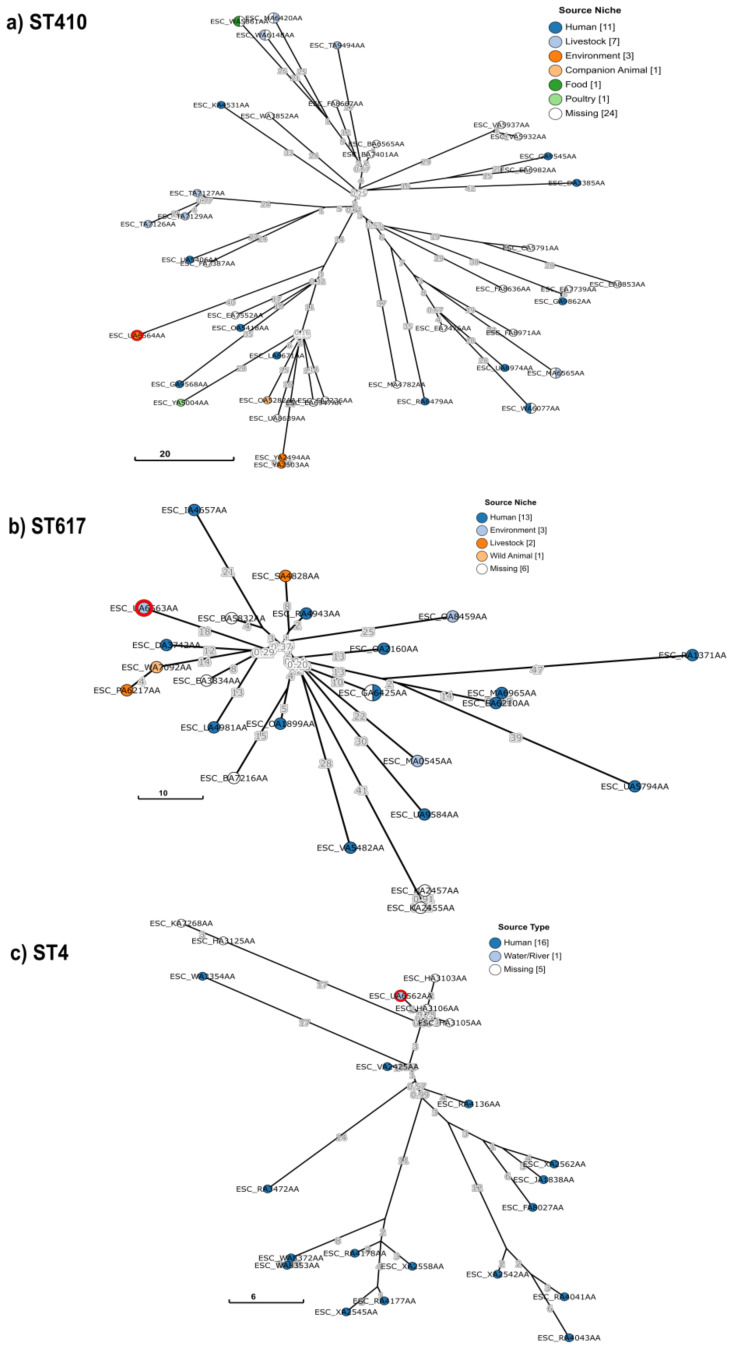
GrapeTree phylogenetic subtrees of the ST410, ST617, and ST4 lineages based on core-genome multilocus sequence typing (cgMLST) distances. The phylogeny was reconstructed using the rapid neighbor-joining (RapidNJ) algorithm and the core-genome MLST (cgMLST) V1 + Hierarchical Clustering (HierCC) V1 scheme from EnteroBase. *E. coli* isolates from this study are highlighted with a red circle. Isolate ID is indicated as the EnteroBase Barcode. The nodes are color-coded according to the isolation source niche as indicated in the key and the numbers in brackets represent the number of isolates. Scale bar indicates the number of cgMLST allelic differences.

Overall, the global collection of ST410, ST617, and ST4 lineages was predominantly depicted by human-derived isolates, and to a lesser extent, by environment-sourced isolates. Of note, a significant number of isolates in each collection are from unknown origin (Supplementary Figures S1–S3). A similar trend was observed during the examination of refined subtrees. All three sequenced *E. coli* were clustered with closely related human-derived *E. coli* isolates (Figure 1a–c).

*E. coli* ADD147/ST410 was placed within a sub-cluster comprising companion animal, environmental, human, and poultry-sourced isolates showed 60–84 cgMLST allelic differences to *E. coli* ADD147 (Figure 1a). Notably, the twelve ST410 isolates belonging to this sub-clade were predicted like ONT:H9 serotype. *E. coli* ADD147 was more closely related to the unknown host source isolate ESC_EA7552AA from Vietnam (60 cgMLST allelic differences) and two human blood-sourced isolates ESC_OA5418AA and ESC_GA9568AA from Canada and Singapore (61–78 cgMLST allelic differences), respectively.

Similarly, *E. coli* ADD167/ST617 was located in a discrete cluster consisting of six isolates sourced from animal, environmental, and human origin showed 28–38 cgMLST allelic differences to *E. coli* ADD167 (Figure 1b). According to cgMLST analysis, the most closely related to *E. coli* ADD167 are the isolate ESC_EA3834AA, recovered from the hospital environment in Germany (26 cgMLST allelic differences), and the human blood-sourced isolate ESC_LA4981AA from the USA (31 cgMLST allelic differences).

Notably, according to EnteroBase and GrapeTree phylogenetic analysis of the global collection of ST4 lineage, *E. coli* ADD183 is the only water-sourced isolate reported. *E. coli* ADD183/ST4 was clustered with human-associated isolates showed 2–31 cgMLST allelic differences to *E. coli* ADD183 (Figure 1c). Furthermore, it was placed into a sub-clade containing five unknown host source *E. coli* isolates from the USA. The most closely related isolates to *E. coli* ADD183 were ESC_HA3106AA, ESC_HA3105AA, and ESC_HA3103AA differing only by two, three, and four cgMLST alleles, respectively.

## 4. Discussion

Aquatic environments are one of the main reservoirs and transmission sources for the dissemination of antibiotic resistance. This study constitutes the first approach for the genomic analysis of three antibiotic-resistant *E. coli* strains from surface water of agricultural drainage receiving raw irrigation water and domestic effluents in northwest Mexico (Sinaloa State). The objective of this genome interrogation was to determine the genetic diversity in terms of virulence features, antimicrobial resistance gene carriage, plasmids, and prophage content and to portray the potential risk that environmental *E. coli* could pose to animal or human health. Although the studied *E. coli* strains were isolated from the same area and period, they showed dissimilar genomic profiles. The resulting differences observed in the phenotypic and genomic profile could be partially explained by (1) the different evolutionary scenarios to which each *E. coli* strain was subjected [44]; (2) the accessory genome dynamics [45]; and (3) the environmental selective pressure imposed by the constant inflow of contaminated agricultural and domestic sewage on the studied agricultural drainage [22]. However, to address this knowledge gap, large-scale genomic epidemiological studies in the One Health framework are needed.

The main concern was the identification of international high-risk clones of *E. coli* carrying multiple acquired antibiotic resistance determinants in the agricultural drainage ditch in the Sinaloa Valley. In this area, intensive agriculture is practiced, and the drained water is subsequently reused for agricultural irrigation, livestock farming, and aquaculture purposes [20]. The “high-risk clone” designation has been used to describe bacterial lineages that enhance the dissemination of antibiotic resistance [46], and a prominent example of this is the ExPEC ST131, a successful globally disseminated clone associated with multiple antimicrobial resistance and enhanced pathogenicity and fitness features [47,48].

In this regard, the MDR TEM-1B-positive *E. coli* strain ADD147 belongs to the widespread ST410 group, a bacterial clone reported worldwide with a high cross-sectorial transmission between animals, humans, and the environment, and bearing diverse ARGs, mostly CTX-M-15, CMY-2, and NDM-5 ESBLs [49,50,51]. Although this isolate was devoid of ESBL genes, it harbored a *sul3*-associated class 1 integron with multiple resistance determinants with the *dfrA12*–*orfF*–*aadA2*–*cmlA1*–*aadA1*–*qacH* gene cassette array, a plasmid-borne integron structure first described in non-typhoidal *Salmonella* [52], and *E. coli* isolates from humans and animals [53,54,55,56]. However, we were not able to link the detected *sul3*-associated integron to a specific plasmid sequence.

The MDR CTX-M-15-positive *E. coli* strain ADD167 belongs to the ST617 lineage, a high-risk clone member of the internationally widespread CC10, which is related to clinical strains found in animal, environmental, food, and human samples worldwide and is predominantly associated with the CTX-M-15 and OXA-1 β-lactamases, and to a lesser extent, with CMY-2 [57,58,59,60,61]. Recent studies have reported the mobile colistin-resistant *mcr*-*1* gene in *E. coli* ST617 from humans and food-producing animals, which threatens the effectiveness of polymyxins, one of the last-resort drug options for treating infections caused by multidrug- and carbapenem-resistant gram-negative bacteria [62,63]. No acquired resistance determinants for the last-resort treatment options were observed.

Based on the investigation of virulence factors, a low abundance of VAGs was predicted in the high-risk clones ADD147 (ST410) and ADD167 (ST617). Alternatively, strain ADD183 (O6:H16) belongs to the ETEC O6 group, the most common ETEC serogroup involved in multiple outbreaks and sporadic infections internationally, and typically carries both enterotoxins [64,65,66]. In contrast to what was reported in ETEC O6 strains [65,67], *E. coli* ADD183 harbored additional nonclassical virulence factors found in ETEC (*eatA*) and ExPEC (*iss*, *kpsE*, and *kpsM_K15*) strains, implying the potential of *E. coli* ADD183 to cause both intestinal and extraintestinal diseases. Therefore, further studies are needed to confirm this hypothesis.

Available information from previous studies shows that clinically sourced colistin-resistant *E. coli* belonging to ST410, ST617, and ST4 lineages have been identified worldwide. In particular, ST410 isolates have been reported in Asia (China and Vietnam), Europe (Italy), and America (Brazil), while ST617 isolates have been described in Asia (China, Korea, and Taiwan), and Europe (France and Italy). Alternatively, ST4 isolates have been reported in Arabia Saudi [68]. It is noteworthy that at present, there is no information regarding the prevalence or distribution of *E. coli* ST410 and ST617 lineages in Mexico, and the unique report of a single human-derived ETEC strain belonging to the ST4 lineage is by Saldaña-Ahuactzi et al. [69].

Although the determination of the origin or direct source was not part of this study, the phylogenetic analysis based on cgMLST elucidated the genetic relatedness and partially gave insight into the clinical relevance of the three *E. coli* strains studied here. Indeed, according to the former analysis, it is most likely that *E. coli* ADD147, ADD167, and ADD183 are human-derived strains that could be introduced into the agricultural drainage by the inflow of untreated domestic wastewater [20,22].

This study had several limitations. The results of the current analysis of a small number of *E. coli* isolates from surface water resources of the selected area cannot be extrapolated to aquatic ecosystems dedicated to agricultural practices at the regional or national level. Nonetheless, despite the limited number of *E. coli* isolates analyzed, our work highlights the need for extensive epidemiological and genomic studies on *E. coli* from the aquatic environment to understand the genetic diversity and the ongoing circulation of multidrug-resistant high-risk clones in the region.

## 5. Conclusions

To date, there is limited information regarding the genomic and molecular data describing the mechanisms underlying antibiotic resistance and virulence properties in *E. coli* from aquatic environments. To the best of our knowledge, this is the first report of genomic profiling of antibiotic-resistant *E. coli* strains from the surface water of agricultural drainage in Mexico. Our study highlights the identification of international high-risk clones ST410 and ST617 of *E. coli* carrying multiple antibiotic resistance determinants, including the widespread CTX-M-15 ESBL, in drained water that is subsequently reused for different practices, such as agricultural irrigation, livestock farming, and aquaculture. Moreover, the presence of enterotoxigenic *E. coli* strains harboring ExPEC-associated virulence factors was also evidenced. These results also underscore the role of surface water as a potential reservoir for critical pathogenic and multidrug-resistant *E. coli* clones and the urgent need for monitoring and tracking these bacterial populations to prevent their ongoing dissemination. Finally, the results of the present study will contribute to antibiotic resistance and virulence surveillance by employing WGS-based methodologies under the One Health concept.

## Figures and Tables

**Table 1:** General features of antibiotic-resistant *E. coli* genomes from surface water of agricultural drainage in northwestern Mexico.

	*Escherichia coli*		
	ADD147	ADD167	ADD183
Length (bp)	4,748,629	4,637,520	4,865,392
GC (%)	50.74	50.94	50.77
Coverage depth (×)	60	60	44
Contigs	594	523	504
Scaffolds (>300 bp)	47	22	6
No. coding sequence (CDS)	4440	4278	4553
Ribosomal RNA (rRNA)	8	8	11
Transfer RNA (tRNA)	81	81	80
Serotype	ONT:H9 ^a^	O101:H10	O6:H16
Phylogroup	C	A	A
Sequence type (ST)	ST410	ST617	ST4
Clonal complex (CC)	CC23	CC10	CC10

^a^ ONT, O-nontypable.

**Table 2:** Phenotypic and genomic profiles of antibiotic-resistant *E. coli* isolates from surface water of agricultural drainage in northwestern Mexico.

Isolate	Antimicrobial Resistance Profile ^a^	Acquired Antimicrobial Resistance Genes ^b^								QRDR Mutations ^b^	Plasmid Replicons ^c^	Virulence Genes ^d^
		β-lactams	Aminoglycosides	Lincosamides	Multidrug Efflux System	Phenicols	Sulfonamides	Tetracycline	Trimethoprim			
**ADD147**	**AM**, **CB**, **CPF**, **CL**, **GE**, **NET**, **NOF**, **STX**	***bla_TEM-1B_***	***aadA1***, ***aadA2***, ***aph(3′)***-***Ia***, ***aac(3)***-***IId***	*lnu(F)*	*mdf(A)*	*floR*, *cmlA1*	*sul2*, *sul3*	*tet(A)*	*dfrA12*	*gyrA*-S83L+D87N; *parC*-S80I; *parE*-S458A	Col(pHAD28), IncFIB(AP001918), IncFIC(FII), IncX1	*gad*, *hra*, *lpfA*, *papC*, *terC*
**ADD167**	AM, CB, CF, CFX, CPF, **GE**, **NF**, NOF, STX	*bla_CTX-M-15_*	*aac(3)*-*IIa*, *aadA5*, *aph(3″)*-*Ib*, *aph(6)*-*Id*	-	*mdf(A)*	-	*sul1*, *sul2*	*tet(B)*	*dfrA17*	*gyrA*-S83L+D87N; *parC*-S80I; *parE*-S458A	Col(MG828), Col(pHAD28)	*astA*, *capU*, *gad*, *iss*
**ADD183**	**CPF**	-	-	-	*mdf(A)*	-	-	-	-	*gyrA*-S83L	ColRNAI, IncFII(pCoo)	*astA*, *capU*, *eatA*, *gad*, *iss*, *kpsE*, *kpsM_K15*, *ltcA*, *ompT*, *terC*, *traT*

^a^ Antimicrobial susceptibility testing by disk diffusion; AM, ampicillin; CB, carbenicillin; CF, cephalothin; CFX, cefotaxime; CPF, ciprofloxacin; CL, chloramphenicol; GE, gentamicin; NET, netilmicin; NF, nitrofurantoin; NOF, norfloxacin; STX, trimethoprim-sulfamethoxazole. A not susceptible interpretation result is highlighted in bold. ^b^ Information extracted from WGS data (ResFinder v4.1, https://cge.cbs.dtu.dk/services/ResFinder/, accessed on 21 November 2021). ^c^ Information extracted from WGS data (PlasmidFinder v2.1, https://cge.cbs.dtu.dk/services/PlasmidFinder/, accessed on 21 November 2021). ^d^ Information extracted from WGS data (VirulenceFinder v2.0, https://cge.cbs.dtu.dk/services/VirulenceFinder/, accessed on 21 November 2021).

**Table 3:** Distribution of intact prophage regions of antibiotic-resistant *E. coli* isolates from surface water of agricultural drainage in northwestern Mexico.

Isolate	Region	Length (kbp)	GC%	CDS	Phage (Hit Genes Counts) ^a^
**ADD147**	1	11.5	48.4	18	*Enterobacteria* phage VT2phi_272 (2)
**ADD167**	1	40.4	50.6	50	*Enterobacteria* phage BP-4795 (9)
	2	15.1	54.5	21	*Escherichia* phage pro483 (18)
**ADD183**	1	40.2	49.9	44	*Enterobacteria* phage P88 (21)
	2	53.7	51.6	45	*Enterobacteria* phage P88 (27)
	3	25.6	49.8	22	*Enterobacteria* phage P4 (9)
	4	10.3	54.2	15	*Escherichia* phage SH2026Stx1 (3)

^a^ Phage with the highest number of CDS most similar to those in the region.

## Data Availability

Publicly available datasets were analyzed in this study. This data can be found here: NCBI BioProject PRJNA715781 (https://www.ncbi.nlm.nih.gov/bioproject/PRJNA715781 accessed on 8 February 2022) and SRA accession numbers: SRX10394959, SRX10394960, and SRX10394961.

## Supplementary Materials

The following supporting information can be downloaded at:https://www.mdpi.com/article/10.3390/microorganisms10030662/s1, Figure S1: Neighbor-joining (RapidNJ) tree based on cgMLST distances of *E. coli* ST410 global collection; Figure S2: Neighbor-joining (RapidNJ) tree based on cgMLST distances of *E. coli* ST617 global collection; Figure S3: Neighbor-joining (RapidNJ) tree based on cgMLST distances of *E. coli* ST4 global collection; Table S1: Metadata associated with *Escherichia coli* ST410 genomes from EnteroBase used for cgMLST subtree phylogeny; Table S2: Metadata associated with *Escherichia coli* ST617 genomes from EnteroBase used for cgMLST subtree phylogeny; Table S3: Metadata associated with *Escherichia coli* ST4 genomes from EnteroBase used for cgMLST subtree phylogeny.
