# Supplementary material for: Genomic Profiling of Antibiotic-Resistant Escherichia coli Isolates from Surface Water of Agricultural Drainage in North-Western Mexico: Detection of the International High-Risk Lineages ST410 and ST617
Source: Microorganisms. 2022 Mar 20;10(3):662. doi: 10.3390/microorganisms10030662 (PMC8948617; doi:10.3390/microorganisms10030662)
Supplement: Supplementary file 1 [file microorganisms-10-00662-s001.zip › microorganisms-1610943-Supplementary.pdf]

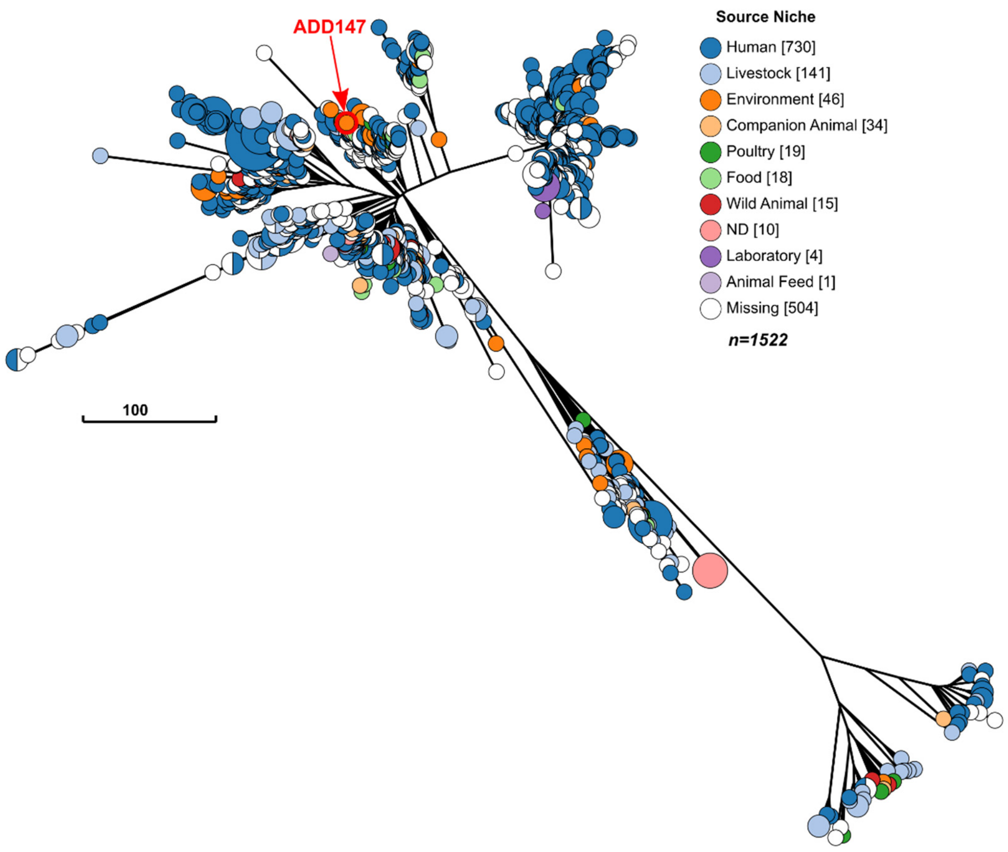

Figure S1. Neighbor-joining (RapidNJ) tree based on cgMLST distances of *E. coli* ST410 global collection. *E. coli* ADD147/ST410 strain described in this study is highlighted with a red arrow and circle. The nodes are color-coded according to the isolation source niche as indicated in the key and the numbers in brackets represent the number of isolates. Scale bar indicates the number of cgMLST allelic differences.

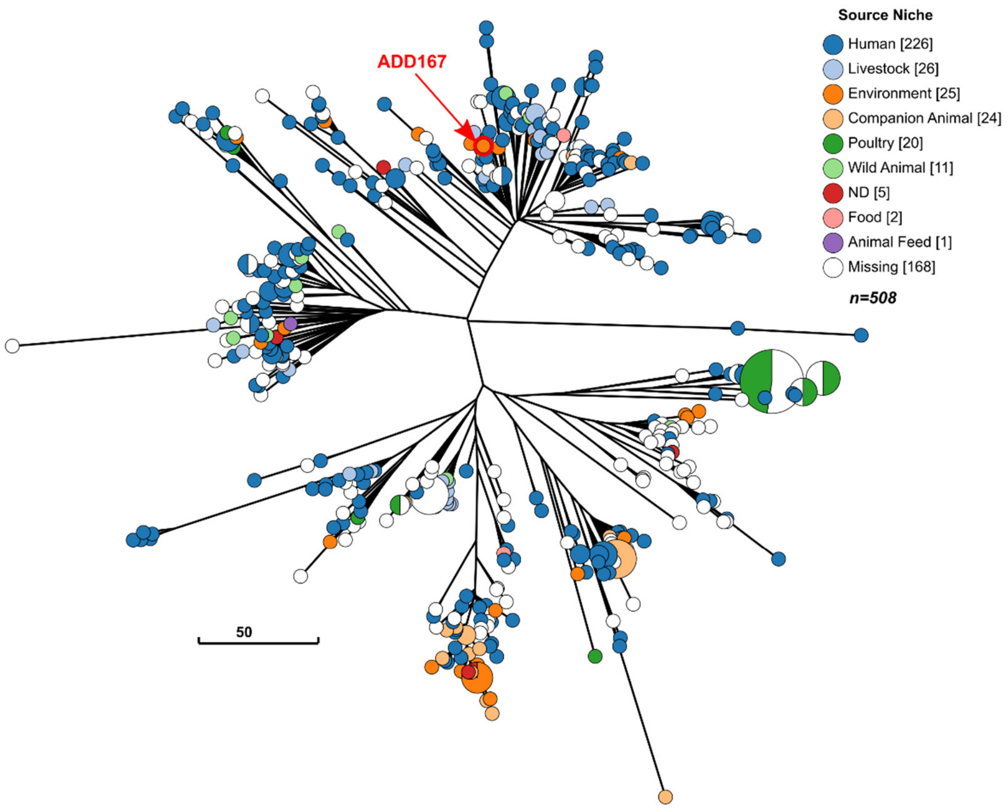

Figure S2. Neighbor-joining (RapidNJ) tree based on cgMLST distances of *E. coli* ST617 global collection. *E. coli* ADD167/ST617 strain described in this study is highlighted with a red arrow and circle. The nodes are color-coded according to the isolation source niche as indicated in the key and the numbers in brackets represent the number of isolates. Scale bar indicates the number of cgMLST allelic differences.

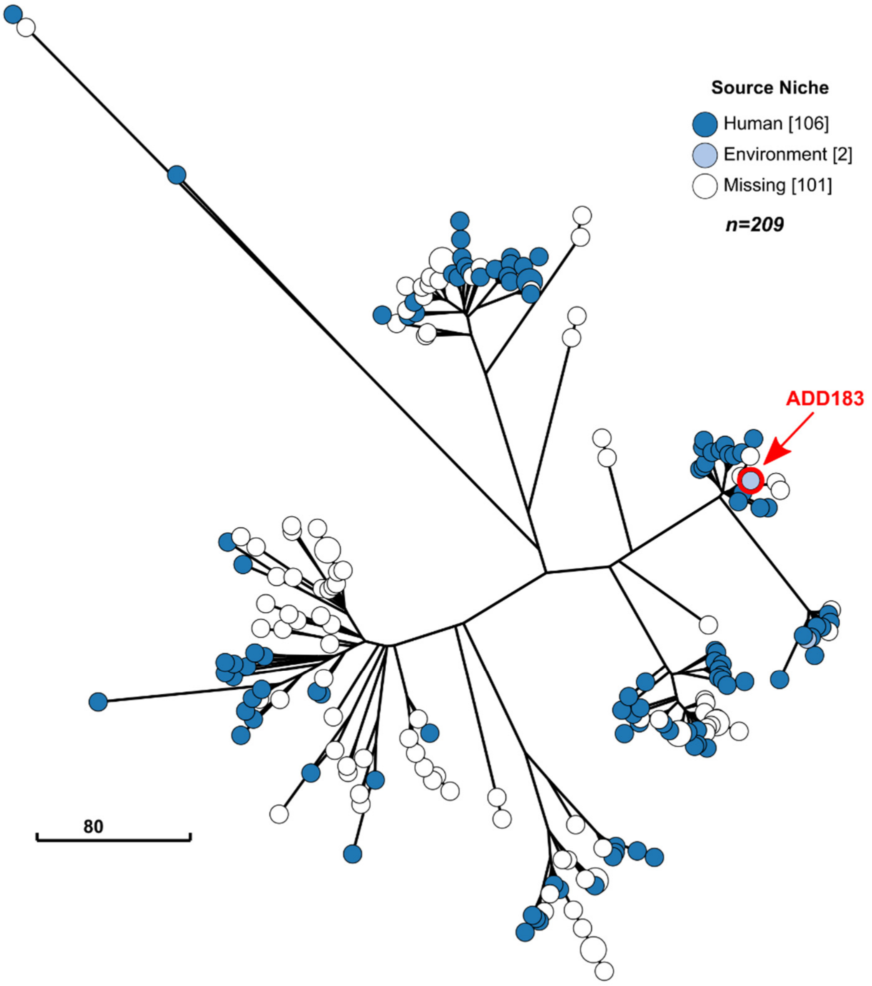

Figure S3. Neighbor-joining (RapidNJ) tree based on cgMLST distances of *E. coli* ST4 global collection. *E. coli* ADD183/ST4 strain described in this study is highlighted with a red arrow and circle. The nodes are color-coded according to the isolation source niche as indicated in the key and the numbers in brackets represent the number of isolates. Scale bar indicates the number of cgMLST allelic differences.
